# Psychological Meanings of Eating Disorders and Their Association With Symptoms, Motivation Toward Treatment, and Clinical Evolution Among Outpatients

**DOI:** 10.5964/ejop.v15i2.1623

**Published:** 2019-06-07

**Authors:** Marie-Pierre Gagnon-Girouard, Marie-Pier Chenel-Beaulieu, Annie Aimé, Carole Ratté, Catherine Bégin

**Affiliations:** a Department of Psychology, Université du Québec à Trois-Rivières, Québec, Canada; b School of Psychology, Université Laval, Québec, Canada; c Department of Psychoeducation and Psychology, Université du Québec en Outaouais, Québec, Canada; d Faculty of Medicine, Université Laval, Québec, Canada; Webster University Geneva, Geneva, Switzerland; Glasgow Caledonian University, Glasgow, United Kingdom

**Keywords:** Bulimia, anorexia, depression, anxiety, motivation, maintenance factors, ambivalence toward treatment

## Abstract

Unlike patients suffering from egodystonic disorders, people with eating disorders sometimes attribute positive meanings to their symptoms, and this attribution process contributes to the maintenance of the disorder. This study aims at exploring psychological meanings of eating disorders and their associations with symptoms, motivation toward treatment, and clinical evolution. Eighty-one adults with an eating disorder (anorexia nervosa, n = 46 and bulimia nervosa, n = 35) treated in a day-hospital program were asked, each week over an 8-week period, to identify the psychological meanings they ascribed to their eating disorder. Avoidance was the most frequently identified meaning, followed by mental strength, security, death, confidence, identity, care, and communication. Avoidance was more frequently mentioned by participants with bulimia than in cases of anorexia. Security and mental strength were associated with less motivation toward treatment. Death was associated with more depressive and anxious symptoms. An exploratory factor analysis showed that these meanings formed three main dimensions: Avoidance, Intrapsychic, and Relational. Findings suggest that psychological meanings associated with eating disorders can be assessed and used as a clinical tool to increase treatment acceptability and effectiveness.

Treating eating disorders (ED) is challenging, notably since a significant number of patients deny being sick, or show ambivalence toward change (Abbate-Daga et al., 2013; Campbell, 2009; Zerbe, 2007). Chronicity and relapse rates have been suggested to be as high as 50% (Keel & Hersog, 2004; Keel & Mitchell, 1997). Treatment dropout rates are worrisome, ranging from 20 to 51% within inpatient samples, and from 29 to 73% in outpatient care (Fassino et al., 2009).

The mere description of eating symptoms severity and frequency do not entirely reflect the complexity of ED (Andersson & Ghaderi, 2006). A review of 24 qualitative studies supports the idea that many patients suffering from anorexia nervosa (AN) value their problematic eating behaviors, viewing them as a way to face personal issues (Espíndola & Blay, 2009). ED symptoms may compensate for low self-esteem, feelings of ineffectiveness, cognitive rigidity, and interpersonal mistrust (Brusset, 1998; Bulik et al., 2005; Mitchell & Bulik, 2006; Palmer, 2008). Since the psychological positive meanings that patients attribute to their ED symptoms may contribute to the maintenance of their dysfunctional eating behaviors and to their reluctance to change, a growing number of authors have proposed a more subjective, patient-centered, approach to better understand the underlying complexity of ED symptoms (Corstorphine et al., 2006; Deaver et al., 2003; Lee & Miltenberger, 1997; Marzola et al., 2016; Serpell et al., 1999; Vitousek, Watson, & Wilson, 1998). Understanding the subjective psychological meanings of ED symptoms may explain patients’ resistances to change, which influences therapeutic alliance and adherence to treatment (Fox, Larkin, & Leung, 2011; McManus & Waller, 1995; Nordbø et al., 2006).

Serpell and colleagues (1999) were among the first to study the psychological meanings of ED. They asked 18 women undergoing treatment for AN to wrote two letters to their ED, one addressing it as a friend and the other as an enemy. Based on these letters, they identified ten themes describing different psychological meanings of AN: *guardian* (providing protection, security, and stability), *control* (giving structure), *attractiveness* (allowing to feel more attractive), *confidence* (increasing self-confidence), *feeling special and different* (allowing to see oneself as superior to others), *ability* (succeeding in something difficult to achieve), *avoidance* (avoiding emotions and distress), *communication* (communicating distress to others), *appearance* and *amenorrhea*. When replicated among patients suffering from bulimia nervosa (BN) (Serpell & Treasure, 2002), two additional themes were identified: *eating without gaining weight* and *coping with boredom*. These results were coherent with the clinical reports demonstrating that, apart from their weight regulation function, bulimic behaviors contribute to the regulation of affect and internal states (Corstorphine et al., 2006; Deaver et al., 2003; Lee & Miltenberger, 1997; Milligan & Waller, 2000; Stickney & Miltenberger, 1999; Waller, Quinton, & Watson, 1995).

In order to get a clearer understanding of patients’ perception of their life with AN, Nordbø and his colleagues (2006) used interactive semi-structured interviews with a focus on the subjective experience of patients. These 90- to 120-minute interviews allowed interviewers to clarify and deepen the perspective of each patient regarding the psychological meanings of their ED. Eighteen women suffering from AN were asked to describe “how it is to have AN?.” Text analyses were then completed based on interviews verbatim. Eight psychological themes were extensively described: *security*, *avoidance*, *mental strength*, *self-confidence*, *identity*, *care*, *communication*, and *death.* This typology overlaps with Serpell’s classification, and is relevant for both AN and BN (Fox, Larkin, & Leung, 2011). Nordbø’s classification was also supported by a systematic review (Espíndola & Blay, 2009).

To date, the efforts devoted to the exploration of the psychological meanings of ED symptoms were based on qualitative studies. Very few quantitative studies have empirically investigated the meanings of ED symptoms, and to our knowledge, only one study examined the association between these self-reported meanings and the clinical profile of patients (Marzola et al., 2016). Based largely on Nordbø’s classification and the systematic review conducted by Espíndola and Blay (2009), Marzola and colleagues (2016) developped a self-report questionnaire designed to assess triggers of AN onset as well as meanings and impact of AN. They reported that psychological meanings ascribed to AN can be regrouped in three independent factors (intrapsychic, relational and avoidance), and that these factors are all associated with eating psychopathology, depressive symptoms as well as personality traits. Factors also showed differential associations with age and motivation to treatment. Considering that the study conducted by Marzola and colleagues (2016) focused only on AN, the inclusion of both AN and BN should be prioritized as most ED treatments are now based on a transdiagnostic approach to ED. Finally, it is important to determine whether psychological meanings ascribed to ED symptoms predict treatment evolution and outcome because differential treatment responses might be expected depending on specific meanings attributed to ED.

In this context, the present study aimed to further study Nordbø and colleagues’ classification (2006) with an open-ended question, in a sample of individuals suffering from AN or BN, and to examine the link between self-reported ED meanings and 1) ED diagnosis (AN or BN), 2) ED symptoms severity and general symptoms (depression and anxiety), 3) motivation toward treatment, and 4) evolution of symptoms and motivation throughout treatment. Results from the present study will also be compared to those recently obtained by Marzola and colleagues (2016) to assess whether the three-factor structure (intrapsychic, relational and avoidance) is robust across clinical samples and if these factors predict symptoms severity, therapeutic motivation, and treatment response.

## Methods

### Participants and Procedure

Eighty-one adult women suffering from AN (*n* = 46) or BN (*n* = 35) participated in the study (aged from 18 to 65 years old). From 2006 to 2010, all women who were admitted to the day-hospital program of the Treatment Program for Eating Disorders at the Centre Hospitalier Universitaire de Québec (Canada) were included. No woman refused to participate in the study, as it was integral part of the treatment program (see procedure for more details). They all received a diagnosis by a psychiatrist specialized in ED who applied DSM-IV criteria to diagnose AN or BN.

### Measures

#### ED and Clinical Symptoms

Eating symptoms were measured using the French version (Leichner et al., 1994) of the Eating Attitude Test (EAT-26), a 26-item self-reported questionnaire (Garner et al., 1982). A higher score indicated greater symptoms and concerns regarding ED. The French version has demonstrated good internal consistency, which was comparable to the original version (Cronbach’s alpha = .90) (Gauthier et al., 2014; Jonat & Birmingham, 2004). Its test-retest reliability in the long term is controversial, mostly because symptoms may vary massively with treatment, which was expected in our study (Banasiak et al., 2001). Depressive symptoms were measured using a French version (Bourque & Beaudette, 1982) of the 21-item Beck Depression Inventory (BDI-II) (Beck, Steer, & Brown, 1996), one of the most widely used psychometric tests for measuring depression. A higher score indicated more severe depressive symptoms. The French version has demonstrated good internal consistency (Cronbach’s alpha = .92) and adequate stability over a 4-month period (Bourque & Beaudette, 1982). Anxiety symptoms were measured by the French version (Freeston et al., 1994) of the Beck Anxiety Inventory (Beck & Steer, 1990), which is a well-known self-reported 21-item questionnaire that assesses physiological symptoms associated with anxiety. Again, a higher score indicated worst physiological symptoms related to anxiety. The French version has demonstrated good internal consistency (Cronbach’s alpha = .93) and adequate test-retest reliability (*r* = .63) (Freeston et al., 1994).

#### Motivation Toward Treatment

Motivation toward treatment was measured with an adapted version of the Treatment Self-Regulation Questionnaire (TSRQ) (Williams, Freedman, & Deci, 1998), an 8-item questionnaire, which was translated in French by our team (double translation process). The original version of the TSRQ assesses reasons for staying in a weight-loss program and this instrument was modified to measure reasons to stay in an ED program. A higher score indicated higher motivation toward ED program. The original version has demonstrated good internal consistency (Cronbach’s alpha ranging from 0.81 to 0.86).

#### Psychological Meanings of ED

The psychological meanings that participants attributed to their ED were documented through an open-ended question: *What does my ED mean to me (what purpose does my ED fulfill)?* Participants could write as many meanings as they wanted.

### Procedure

The detailed study protocol was reviewed and approved beforehand by the ethic committee of the hospital where the study took place. On the first day of the program, a doctoral student informed the patients about the purpose and procedure of the study. All participants were invited to participate in the study and were informed that, during the treatment program, time would be reserved to complete the questionnaires. If they agreed to participate, they signed an informed consent form approved by the ethic committee of the hospital. Then, questionnaires were filled in by participants every Friday morning for the entire length of the 8-week treatment program. All questionnaires were completed in the same order as presented in the measures section. Accordingly, the question regarding psychological meanings of ED was completed at the end of the protocol each week.

### Data Analysis

#### Classification Procedure

Based on Nordbø and colleagues’ classification (Nordbø et al., 2006), we categorized participants’ statements into ED psychological meanings. Classification procedure was based on the model proposed by Taylor-Powell and Renner (2006) and consisted of four steps: 1) a priori description of themes, 2) preliminary reading of the data, 3) improvement of code definitions, and 4) coding. This procedure allowed preserving the nature of the data generated by participants while allowing the replication of previous structures (Nordbø et al., 2006). Based on Nordbø and colleagues’ denominations, we first defined an a priori list of eight themes representing proposed psychological meanings. Two independent raters with ED clinical experience made a preliminary reading of all the available data to ensure that no additional theme was needed, after which the definitions of themes were refined, leading to a detailed description of all meanings (summarized in Table 1). For example, at this step, it was proposed to add the theme *Death,* to include statements related to self-destruction in general. The two raters used the final version of the typology to code all participants’ statements. Inter-raters agreement was tested on a sub-sample of 15 participants at the beginning of classification and at mid-process.

**Table 1:** Brief Description of Meanings Based on the Classification From Nordbø and Colleagues (Nordbø et al., 2006)

Function	Description	Examples from the present study
Security	A way to obtain a sense of stability and security.	"To keep my body and life under control"
Avoidance	A way to avoid negative emotions and experiences.	"To regulate my unease, my emotions" "To avoid my responsibilities"
Mental strength	A way to get an inner sense of mastery and strength.	"To make me feel strong" "To give me the feeling that I am more disciplined"
Confidence	A way of feeling worthy of compliment and acknowledgment.	"Others have a more positive image of me" "To make me feel more desirable"
Identity	A way of creating a different identity or personality.	"To be different" "To become someone else"
Care	A way of eliciting care from other people.	"To be closer to my mother, there is more support and complicity between us"
Communication	A way of communicating difficulties to other people.	"To express my distress and unease"
Death	A way of starving to death, of serving self-destructive purposes	"To disappear" "To punish myself" "To destroy myself"

#### Statistical Analyses

Statistical analyses were performed with the SPSS Statistical software (version 19.0). For each participant, a score from 0 to 8 was assigned for each meaning, based on the weekly identification of the meaning. A score of 0 was attributed if the meaning was never mentioned by the participant whereas a score of 8 was attributed if the meaning was mentioned every week of the program. For example, a participant who identified only *avoidance* from week 1 to 8 and *security* from week 2 to week 8 obtained a score of 8 on the *avoidance* meaning, 7 on the *security* meaning, and 0 for all the other meanings. Consequently, for each participant, each meaning had an endorsement score (from 0 to 8) that represented the frequency of its adoption in the participant’s responses.

*t*-tests were computed to compare ED diagnosis groups (AN vs. BN) on the eight meanings endorsement’s scores. Pearson’s coefficients were computed to assess the relationship between meanings endorsement scores on one hand, and the clinical symptoms, motivation toward treatment and evolution throughout treatment, on the other hand. The evolution of symptoms and motivation throughout treatment was calculated as the difference between the mean score at the end of treatment and the mean score at baseline.

Pearson’s correlation analyses were also computed to assess the relationships between the eight meanings endorsement scores. An exploratory factorial analysis was also performed with the Varimax rotation method in order to obtain a more easily interpretable structure. The Kaiser-Meyer-Olkin (KMO) indicator was used to measure the sampling adequacy, with a KMO of 0.7 considered as acceptable. A confirmatory factorial analysis was computed to verify the fit of the factorial structure in the data from the sample. Indices of adjustment were the chi-square test, the Comparative Fit Index (CFI), and the Root Mean Square Error of Approximation (RMSEA). A chi-square value close to zero and a chi-square p-value greater than 0.05 indicate a good fit, as well as a CFI value of 0.90 or greater and a RMSEA of 0.06 or less.

## Results

### Classification

Inter-raters agreement for classification of statements was excellent, with a kappa coefficient of 0.91. Most statements (79.68%) were classified within Nordbø and colleagues’ classification (2006). Statements that did not fit into any category were labelled “others.” These statements were either non-psychologically oriented or very vague (for example, I don’t know why). No consistent new theme emerged from these statements.

Mean scores for each meaning are presented in Table 2. A*voidance* was the most frequently identified meaning, followed by *mental strength*, *security*, *death*, *confidence*, *identity*, *care*, and *communication.*

**Table 2:** Means and Standard Deviations for Meanings

Function	Total (*n* = 81)		AN (*n* = 46)		BN (*n* = 35)		Difference between AN and BN	
	*M*	*SD*	*M*	*SD*	*M*	*SD*	*t*	*d*
Avoidance	3.12	2.19	2.59	1.73	4.00	2.47	-2.884**	0.66
Mental strength	1.49	1.93	1.78	2.17	1.14	1.56	1.478	0.34
Security	1.11	1.44	1.13	1.54	1.00	1.19	0.415	0.09
Death	0.62	0.99	0.65	1.40	0.63	1.00	0.084	0.02
Confidence	0.52	1.15	0.52	1.09	0.57	1.27	-0.189	0.04
Identity	0.46	0.90	0.59	1.02	0.31	0.72	1.408	0.32
Care	0.43	1.25	0.48	1.38	0.40	1.12	0.274	0.06
Communication	0.33	0.99	0.28	0.91	0.43	1.12	-0.647	0.15

*Note.* AN = anorexia nervosa; BN = bulimia nervosa.

**р < .01.

### ED Diagnosis

Mean scores for psychological meanings according to ED diagnosis are presented in Table 2. Participants suffering from BN endorsed significantly more often the *avoidance* meaning than participants suffering from AN (*d* = 0.66). Participants suffering from AN mentioned more often the *mental strength* and *identity* meanings than participants with BN, and even if the differences did not reach significance, the effect size were moderate (*d* = 0.34 and 0.32). No difference was noted for other meanings. For participants with BN, *avoidance* was endorsed 3.5 times more than the second most frequent meaning which was *mental strength*, whereas for participants with AN, the difference between *avoidance* and *mental strength* was of lesser magnitude (*avoidance* 1.5 times more frequent than *mental strength*).

### Short-Term and Long-Term Links With Clinical Profile

Correlations between meanings and symptoms, motivation, and their evolution are presented in Table 3. *Death* was positively associated with depressive and anxiety symptoms, meaning that the more a participant endorsed the *death* meaning, the more this participant reported being depressed and anxious at the beginning of treatment. *Security* and *mental strength* were negatively associated with motivation toward treatment, meaning that the more a participant endorsed these meanings, the less she reported being motivated at the beginning of treatment. There was no other significant correlation between meanings and ED symptoms. There was no significant association between meanings and the evolution of symptoms and motivation throughout treatment.

**Table 3:** Pearson’s Coefficients for Relations Between Meanings and the Severity of Eating Symptoms, Depression, Anxiety, Motivation, and Their Evolution From Pre-Treatment to Post-Treatment

Symptoms/function	Security	Avoidance	Mental strength	Confidence	Identity	Care	Communication	Death
Eating symptoms	.095	-.052	.153	.088	.061	.041	.006	.198
Depression	.073	.034	-.099	-.163	-.014	-.070	-.119	.315**
Anxiety	.139	.041	-.175	.002	.031	.011	.046	.328**
Motivation	-.249*	.115	-.241*	.073	-.006	.123	.129	-.028
Changes in eating symptoms	.147	.039	.077	.048	.241	.008	.063	.181
Changes in depression	-.060	.157	-.003	.027	.109	.066	.038	.238
Changes in anxiety	-.001	.114	.246	-.022	.091	-.127	-.142	.050
Changes in motivation	-.034	-.013	.077	-.169	-.218	-.140	-.121	.069

*р < .05. **р < .01.

Correlations between meanings are shown in Table 4. *Confidence*, *care* and *communication* were positively associated with one another (moderate coefficients), as were *confidence, identity,* and *mental strength* (strong coefficients).

**Table 4:** Pearson’ Coefficients for Correlations Between Meanings

Function	1	2	3	4	5	6	7	8
1. Security	-	-.028	.175	-.010	.150	-.040	.080	-.129
2. Avoidance		-	-.071	-.062	-.072	.144	.151	.059
3. Mental strength			-	.275*	.317**	.040	.127	-.142
4. Confidence				-	.276*	.437**	.599**	-.158
5. Identity					-	.164	.204	-.193
6. Care						-	.775**	.015
7. Communication							-	-.010
8. Death								-

*р < .05. **р < .01.

An exploratory factor analysis yielded three factors, based on the presence of three eigenvalues greater than 1, for a percentage of explained variance of 62% (Table 5). The KMO for the factorial structure was 0.62, which shows a poorly fitted model. Based on the structure proposed by Marzola and colleagues (2016), the three factors were named *Avoidant* (*avoidance*), *Relational* (*confidence, care*, and *communication*), and *Intrapsychic* (*mental strength, identity*, and *death*). *Security* loaded significantly on both the *Avoidant* and *Intrapsychic* factors, and it was considered part of the Intrapsychic factor, to be coherent with Marzola and colleagues’ (2016) typology. Moreover, the magnitude of the correlation coefficients was more important between security and the subscales of the intrapsychic factor (*r* = .129 to .175) than the avoidant one (*r* = .028). The confirmatory factor analysis revealed a better adjustment to the data (χ² = 21.79, *p* = .295; CFI = 0.97, RMSEA = 0.042). This confirmatory factor analysis suggests that the replication of this analysis in a larger sample could validate it, notably with a sample of at least 10 subjects per variable, as previously suggested (Hair, 1998). Correlations between factors and symptoms, motivation, and their evolution were also performed. There was no significant association (*r* ranging from −0.04 to −0.18).

**Table 5:** Meanings’ Loadings Following the Exploratory Factor Analysis

Function	Avoidance	Relations	Intrapsychic
Security	**.55**	-.16	**.57**
Avoidance	**.81**	.16	-.22
Confidence	-.22	**.74**	.31
Care	.12	**.88**	-.06
Communication	.16	**.91**	-.08
Mental strength	.02	.12	**.69**
Identity	-.04	.24	**.66**
Death	.15	.04	**-.54**

## Discussion

The present study aimed at exploring the subjective psychological meanings that ED patients attribute to their ED, based on a previous typology by Nordbø and colleagues (2006), using a simple self-reported statement instead of interviews. This study adds to existing literature by exploring the links between self-reported ED meanings and ED diagnosis (AN or BN), ED and general symptoms severity, and motivation toward treatment, as well as evolution of symptoms, and motivation throughout treatment, in a sample of women suffering from either AN or BN.

First, the very high inter-rater agreement (*K =* 0.91) as well as the high percentage of statements that could be categorized (79.68%) showed that the classification from Nordbø and colleagues (2006) has clinical significance among patients suffering from AN and BN, and can be used to classify simple self-reported statements. In previous studies, the meanings were identified through carefully conducted interactive semi-structured interviews, which greatly deepened the understanding of underlying meanings attributed to ED by participants, but which were costly and required a lot of time and resources. The present study replicates this typology with a simple self-reported question repeated throughout treatment, which is significantly less costly and complex. Findings give empirical support to this method of assessing psychological meanings of ED in clinical settings.

Participants in this study identified mainly *avoidance, mental strength,* and *security* as the primary meanings of their disorder, which is similar to previous studies’ findings (Nordbø et al., 2006; Serpell & Treasure, 2002; Serpell et al., 1999). When ED diagnosis was taken into consideration, it was found that the adoption of disturbed eating behaviors to soothe or avoid aversive internal states, as described by the *avoidance* meaning, was more frequently reported by participants suffering from BN than by those with AN. These results are in concordance with the literature, suggesting that binge eating may serve to escape or temporarily reduce negative affects such as anxiety, anger, irritation, depression, and boredom (Deaver et al., 2003; Heatherton & Baumeister, 1991; Kaye et al., 1986; McManus & Waller, 1995; Milligan & Waller, 2000; Stickney & Miltenberger, 1999; Waller, Quinton, & Watson, 1995), by shifting the attention focus on food, while purging behaviors can serve to reduce guilt and worry associated with binge eating (Corstorphine et al., 2006; Kaye et al., 1986; Milligan & Waller, 2000).

Among participants with AN, the *security* and *mental strength* meanings were the most frequently endorsed meanings, although they did not appear to be as common as in previous studies, avoidance being the most frequent meaning (Nordbø et al., 2006; Serpell et al., 1999; Vitousek, Watson, & Wilson, 1998). Such dissimilarity might be related to our sample composition, in which both subtypes of AN patients (restricting and binge-eating/purging) were included. *Security* and *mental strength* are possibility more prevalent in purely restrictive AN patients, who are known to show high self-control. Control over one’s body and food could artificially compensates for feelings of ineffectiveness and low self-esteem, as an attempt to internally control external reality (Chassler, 1994; Gabbard, 2000; Vitousek, Watson, & Wilson, 1998).

Considering the aggregation of meanings into three main dimensions, results from our study not only replicate the three-factor structure proposed by Marzola and colleagues (2016) but the theoretical content of our factors is similar to the one obtained by this research team. The avoidance factor represents a strategy for escaping negative emotions and experience. The relational factor represents a way of communicating patient’s own messages via problematic symptoms (ex. a way of communicating difficulties to other people, of eliciting care from other people, of feeling acknowledged). The intrapsychic factor is more oriented toward patients own motives and internal world (ex. a way of obtaining a sense of stability and security, of getting an inner sense of mastery and strength, of creating a different identity or personality). In the present study, the three factors were not related to ED symptoms. Nevertheless, subscales from the intrapsychic factor were associated with greater psychopathology (*death*) and less motivation toward treatment (*mental strength and security*). These results are similar to those obtained by Marzola and colleagues (2016), who found more depression and less motivation in relation with the intrapsychic factor. Intrapsychic motives may be related to less favorable prognostic (Marzola et al., 2016). It is however important to note that *death* being part of our intrapsychic factor was a novel finding, and this should be replicated. The absence of difference within the three factors regarding ED symptoms in the present study might be explained by the fact that we used a rather general measure of eating problems, instead of a more specific one, as Marzola and colleagues (2016) did*.* As such, they found different associations between the factors and ED problems depending on the facets measured. Still, they did not find associations between their factors and BMI, duration of illness as well as duration of treatment/psychotherapy. According to them, the independence of ED meanings from duration of illness and treatment supported their results since it ensured that psychological meanings were not contaminated by illness or over-rationalization following treatment.

From a clinical point of view, our study underlines the relevance of considering the subjective reality of ED patients, rather than only describing EDs as observable and discrete behaviors. As Nordbø and colleagues (2006) mentioned, patients describe the psychological meanings of ED as precipitating, contributing and maintaining their disorder. Adding interventions specifically targeting psychological meanings of EDs to existing treatment could increase treatment acceptability and effectiveness, especially if these interventions address ED’s benefits such as a greater sense of security, identity and mental strength. For the patient, it could carry a feeling of being more fully understood (as the therapist would understand the meaning of problematic behaviors) and could give sense to the healing process, which helps developing the therapeutic alliance, as well as ensuring motivation and adherence to treatment (Fox, Larkin, & Leung, 2010; Nordbø et al., 2006). Furthermore, by targeting the prominent ED meanings for each patient, interventions would be more personalized and adapted to the patient discourse, which may reduce the heterogeneity of the clinical response. Finally, considering that ED develops at a relatively young age, it would be very interesting for future studies to investigate ED meanings among teenagers to validate the different themes that we have found and their frequency among those patients. This will support future interventions on ED meanings with adolescents.

Although the present study has a number of strengths (a single question to assess ED psychological meanings, distinction between AN and BN, reference to a theoretical framework), results must be interpreted in light of some limitations. First, considering that all patients were recruited at their admission session of a day hospital program in a specialised ED unit, we cannot ensure that their ED diagnosis (AN or BN) remained stable throughout the study as diagnosis crossover may have happened. Second, although the sample size was larger than previous studies, it was still not large enough to examine subtypes of AN. Further studies should test these associations within sub-samples of individuals with AN (restrictive or binge-eating/purging), BN and BED separately, with more refined measures of eating symptoms. Finally, given the correlational nature of the present study, the direction of causality between ED psychological meanings and clinical symptoms, motivation, and their evolution cannot be assumed.

In sum, the present study adds to previous empirical investigations by showing that ED psychological meanings can be assessed rigorously by a simple self-reported statement, which is less expensive and time-consuming than semi-structured interviews. In addition, findings suggest that the inclusion of explicit interventions specifically targeting psychological meanings of ED could increase treatment acceptability and effectiveness.
